# StructureNet: Physics-Informed Hybridized Deep Learning Framework for Protein–Ligand Binding Affinity Prediction

**DOI:** 10.3390/bioengineering12050505

**Published:** 2025-05-10

**Authors:** Arjun Kaneriya, Madhav Samudrala, Harrish Ganesh, James Moran, Somanath Dandibhotla, Sivanesan Dakshanamurthy

**Affiliations:** 1 College of William and Mary, School of Computing, Data Sciences & Physics, Williamsburg, VA 23185, USA; 2 College of Arts and Sciences, The University of Virginia, Charlottesville, VA 22903, USA; 3 VCU Life Sciences, Virginia Commonwealth University, Richmond, VA 22043, USA; 4 College of Arts and Sciences, Georgetown University, Washington, DC 20057, USA; 5 College of Engineering and Computing, George Mason University, Fairfax, VA 22030, USA; 6 Department of Oncology, Lombardi Comprehensive Cancer Center, Georgetown University Medical Center, Washington, DC 20007, USA

**Keywords:** protein–ligand binding affinity, machine learning, drug discovery, structure-based graph neural network

## Abstract

Accurately predicting protein–ligand binding affinity is an important step in the drug discovery process. Deep learning (DL) methods have improved binding affinity prediction by using diverse categories of molecular data. However, many models rely heavily on interaction and sequence data, which impedes proper learning and limits performance in de novo applications. To address these limitations, we developed a novel graph neural network model, called StructureNet (structure-based graph neural network), to predict protein–ligand binding affinity. StructureNet improves existing DL methods by focusing entirely on structural descriptors to mitigate data memorization issues introduced by sequence and interaction data. StructureNet represents the protein and ligand structures as graphs, which are processed using a GNN-based ensemble deep learning model. StructureNet achieved a PCC of 0.68 and an AUC of 0.75 on the PDBBind v.2020 Refined Set, outperforming similar structure-based models. External validation on the DUDE-Z dataset showed that StructureNet can effectively distinguish between active and decoy ligands. Further testing on a small subset of well-known drugs indicates that StructureNet has high potential for rapid virtual screening applications. We also hybridized StructureNet with interaction- and sequence-based models to investigate their impact on testing accuracy and found minimal difference (0.01 PCC) between merged models and StructureNet as a standalone model. An ablation study found that geometric descriptors were the key drivers of model performance, with their removal leading to a PCC decrease of over 15.7%. Lastly, we tested StructureNet on ensembles of binding complex conformers generated using molecular dynamics (MD) simulations and found that incorporating multiple conformations of the same complex often improves model accuracy by capturing binding site flexibility. Overall, the results show that structural data alone are sufficient for binding affinity predictions and can address pattern recognition challenges introduced by sequence and interaction features. Additionally, structural representations of protein–ligand complexes can be considerably improved using geometric and topological descriptors. We made StructureNet GUI interface freely available online.

## 1. Introduction

Predicting protein–ligand binding affinity is a key component of the drug discovery process. Binding affinity allows us to understand the stability between a drug and its target receptor. This plays a large role in the potency, efficacy, and therapeutic potential of a drug candidate [1]. In addition, accurate drug–target binding affinity predictions significantly reduce drug development times and costs, which remains a challenge today. Traditional scoring functions fail to accurately estimate binding affinity and struggle in lead optimization and de novo design [2]. Because of this, approaches to binding affinity prediction have shifted to machine learning (ML) and deep learning (DL) models in recent years. ML and DL algorithms have found great success in predicting binding affinities through their pattern recognition abilities. These algorithms are capable of producing faster, more accurate predictions than physics-based and empirical scoring functions [3].

DL models displayed particularly high performance in binding affinity prediction [4,5]. Stable and robust DL models are able to achieve high predictive performance while emphasizing scalability and efficiency. Convolutional neural networks (CNNs) have led the implementations of deep learning in drug–target affinity (DTA) prediction, representing features from binding sites in either 1D, 2D, or 3D. In DeepDTA, Öztürk et al. used a CNN trained and tested on 1D representations of drugs (protein sequences) and targets (SMILES strings) [6]. In K_DEEP_, Jimenez et al. used a 3D-CNN to extract features from protein–ligand complexes partitioned into small cubic grids, with different voxels modeling areas of different pharmacophoric properties [7]. Another subsection of DL models, graph neural networks (GNNs), have also been employed for DTA prediction to better represent the structural and topological features of protein–ligand complexes. In GIGN, Yang et al. trained and tested a geometry-aware GNN on 3D structures and intermolecular interactions represented as graphs [8]. Planet, developed by Zhang et al., also uses a combination of structural and interaction descriptors to achieve high accuracy in binding affinity predictions [9].

Despite their predictive success, the aforementioned DL models tend to generalize poorly and underperform on unseen de novo data. There is evidence that neural networks using the entire protein–ligand complex as input do not maximize the information gained from protein–ligand interaction features, since the same models trained on individual protein or ligand structures perform equally well [10,11]. Architectures that use overly complex inputs often fail to learn from interaction descriptors and only learn from structure-based ones. Therefore, no real relationship between descriptor type (sequence vs. structure vs. interaction) and increased model performance has been found for binding-affinity-predicting deep learning (BAPDL) models [12]. Furthermore, no category of features has been proven to drive model accuracy and robustness purely through learning as opposed to memorization. In fact, training DL models on data that do not capture structural information may even impair their performance, particularly in de novo applications [13,14]. Frameworks that ignore the intermolecular interactions stored in the protein–ligand bound state have shown equal or higher predictive and generalizability power than those that consider it [15,16]. Because of this, it is within reason to speculate that BAPDL models are simply memorizing patterns within feature spaces and not learning from them when given complex combinations of sequence- and interaction-based data. Although the inclusion of interaction and sequence features has increased model accuracy, it is for reasons counterintuitive to using a deep learning model. The high-performance metrics of these models are only present during the training/testing phase and tend to nosedive when the models are tested on unseen data. Because of these limitations, there is a growing interest in exploring BAPDL approaches that do not rely on sequence or interaction features. Since structural features model physical characteristics, which are inherent in all molecules, they offer routes for more generalizable models with less limitations. Past BAPDL models have included structural features, but few studies exist that exclusively use structural descriptors without any interaction or sequence features [17,18,19,20,21,22,23].

In this study, we developed a structure-based deep learning model to predict binding affinity and offer an alternative approach to feature representation that addresses the described limitations. Our model, named StructureNet, exclusively uses structural features of protein–ligand complexes to make informative binding affinity predictions. We include unique geometric calculations in the form of Voronoi tessellations along with traditional biochemical data to improve molecular representation. StructureNet represents the protein-binding pocket and ligand as two separate graphs and generates predictions using a GNN-based ensemble deep learning architecture. StructureNet exhibits moderate predictive power and robustness as a standalone model and was able to differentiate between different classes of ligands in external validation testing. Furthermore, metrics generated from drug predictions showed that StructureNet effectively learns from patterns in protein–ligand complexes without simply memorizing training data. To our knowledge, StructureNet is one of only two traditionally structured GNNs that uses a deep learning approach to predict binding affinity using exclusively structural features (see Section 3.6).

## 2. Materials and Methods

### 2.1. Dataset

During standard training and testing procedures, we used the “refined” and “general” sets from the PDBBind v2020 database for protein–ligand complexes. The refined set provides experimental data for 5316 protein–ligand complexes, including files for each complex’s protein molecule, ligand molecule, and protein-binding pocket (File S1). The general set houses the rest of the protein–ligand complexes and represents a broader, more diverse range of experimental binding affinities (File S2). Files S1 and S2 can be found at: “https://github.com/sivaGU/StructureNet (accessed on 27 January 2025)”. For each complex in the refined set, PDBBind provides the complex’s PDB code; file resolution; release year; reference; corresponding ligand name; and two measures of binding affinity, the *K_d_* dissociation or *K_i_* inhibition constant, and the negative base-10 logarithm transformation of *K_d_*/*K_i_* (−log(*K_d_*/*K_i_*). Note that the “/” denotes the use of *K_d_* (the dissociation constant) or *K_i_* (the inhibition constant). In our models, we predicted protein–ligand binding affinity as the (−log(*K_d_*/*K_i_*) for normalization purposes. In the general set, the IC_50_ value for a protein–ligand complex may be listed in place of *K_d_*/*K_i_*. Thus, complexes with a reported IC_50_ value were filtered out from the dataset.

For further training and testing, we merged the refined and general sets to create a separate “combined set”. The refined and general sets have no protein–ligand complexes in common. For standard training and testing procedures, the ratio of training to testing data was determined and specified during K-Fold Cross Validation (see Section 2.6). The assignments of complexes to either the training or testing set were randomized for every run. In our first external validation phase, we used the DUDE-Z dataset, a newer version of the Database of Useful Decoys (DUDE) [24]. DUDE-Z contains files for 43 receptors along with thousands of active ligands and artificial decoys that closely mimic the actives in structure. We converted ligand files from .mol2 to .pdb for ease of use with our model. The DUDE-Z database contains pre-docked ligands and decoys with the receptors, which is a prerequisite for later versions of the model.

### 2.2. Protein–Ligand Graph Creation from PDB Complex Files

For input into the GNN, we represent the structural features of the protein-binding pocket and ligand in graphs using the NetworkX Python library (version 3.1), which streamlines the creation and manipulation of graph-based data methods. Graphs for both molecules are constructed separately but concatenated prior to training and testing. Nodes and edges in each graph held information about atomic and bond-related properties, respectively. In the protein-binding pocket graph, a distance cutoff from the ligand was employed to determine which protein atoms would be included in the protein graph. Two arrays were separately constructed for each complex representing molecular-level features of either the protein-binding pocket or the ligand. These features were stored in the graphs as graph-level features and were later combined with the node and edge feature embeddings in the GNN during training and testing.

### 2.3. Feature Extraction and Molecular Representation

**Figure 1:**
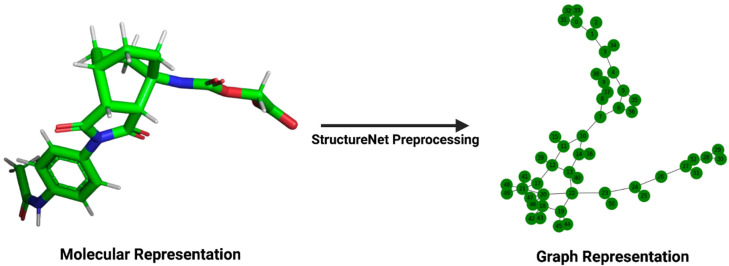
Transformation of a molecular structure into its graph representation. The left panel depicts the structure of a ligand, viewed in PyMOL (version 2.5.5), from its .pdb file in the PDBBind v2020 refined set. The right panel depicts the same ligand’s graph representation for input into StructureNet.

Our feature extraction process used the RDKit library to read in and sanitize the molecules, extract their respective features, and represent them in graphs (Figure 1). After a molecule is imported, a custom function is used to fully sanitize and ensure its chemical validity. The sanitization process checks for common chemical incompatibilities and certifies that the structure of the file has been correctly interpreted by RDKit. Descriptor functions were then used to extract various molecular features for both nodes and edges, after which both subgroups of features were added to their respective arrays. Blank NetworkX graphs were populated with the calculated node and edge features before being converted to data types compatible with the PyTorch Geometric (version 2.6.1) GNN constructors.

The graph representations of the protein-binding pocket and ligand incorporated fourteen node features and six edge/bond features (Table 1). Feature values appeared either as integer values, one-hot-encodings, or binary (0 if not, 1 if yes). Even though RDKit’s descriptor library is expansive, it does not directly calculate several important features pertaining to binding affinity. Features extracted through custom functions are denoted with a “*”. Some of these functions relied on RDKit to an extent, while others were fully custom-made.

Notably, we chose to include Voronoi diagrams and spherical harmonics calculations in our feature representation (Table 1). Voronoi tessellation breaks up space into regions based on proximity to a given set of objects. The area in each region is closer to its object than any other point in the space. For a set of points $\left\{p_{i}\right\}$ in space,

(1) $$V(p_{i})=\{x\in R^{3}|\;||x-p_{i}||\leq ||x-p_{j}||\;\forall j\neq i\;\}$$

where *V*(*p_i_*) is the Voronoi cell for a point *p_i_*. We apply Voronoi tessellation to the protein-binding pocket and ligand such that each atom represents a point. Each structure is thereby partitioned into regions associated with their individual atoms. The main objective for including Voronoi was to better capture the local geometric contexts around each molecule. By describing precise arrangements of atoms, Voronoi data can detail potential binding site characteristics like residue distribution and pocket geometry [25]. For further structural analysis, we include spherical harmonics calculations, which are used to describe surfaces of round objects. Our implementation of spherical harmonics followed

(2) $${Y_{l}}^{m}(\Theta ,\varphi )=\sqrt{\frac{(2l+1)(l-m)!}{4\pi (l+m)!}}e^{im\varphi }{P_{l}}^{m}(cos\theta )$$

for a degree *l*, order *m*, and associated Legendre polynomials *P_l_^m^*. We included spherical harmonics calculations to describe the local geometric symmetry around atoms. Geometrically, spherical harmonics highlight areas of structural similarity, like symmetry (or asymmetry). Since spherical harmonics calculations were represented as node features, they describe angular patterns at an atomic level, enhancing descriptive potential. These calculations can also give StructureNet insight into local electron densities and possible binding sites. Applications of spherical harmonics calculations have even improved virtual screening techniques when compared to traditional shape-based methods [26].

**Figure 2:**
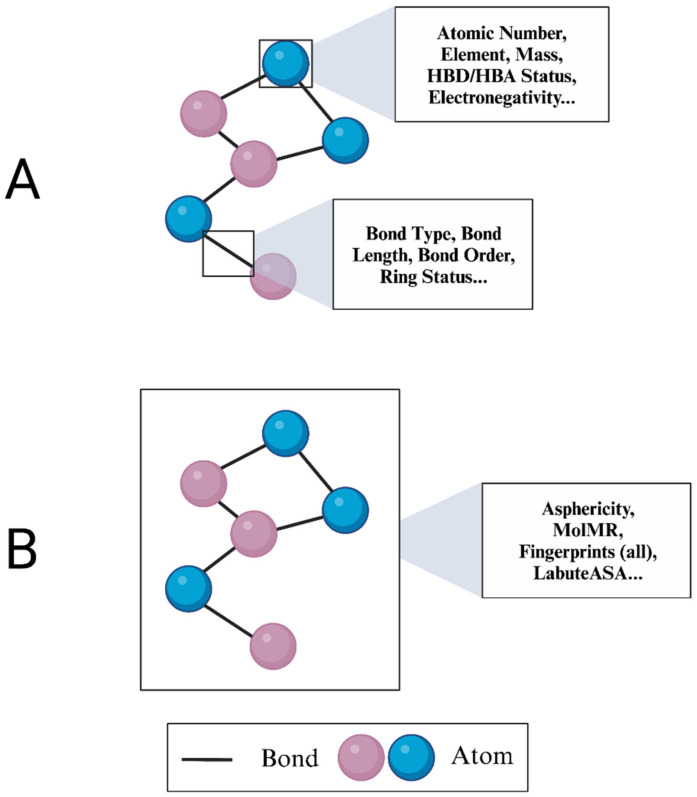
Visualization of node, edge, and graph feature calculations. (**A**) Nodes and edges in StructureNet graphs represent atom and bond-level properties, respectively. (**B**) Graph-level features in StructureNet graphs represent global molecule-level characteristics.

In addition to atom- and bond-level features, we extracted molecular features from the ligand and protein-binding pocket. Feature arrays were separately constructed to detail these molecule-level attributes and capture larger patterns not evident in node and edge features (Figure 2). Molecular feature arrays for both the protein-binding pocket and the ligand included 70 molecular descriptors mainly describing surface area, topology, and hydrophobicity (Table 2). These arrays were treated as graph-level features in their respective protein-binding pocket and ligand graphs. An asterisk (*) denotes the use of a feature in only one of the two feature arrays produced for each complex.

We performed min–max normalization on all features to ensure that the ranges were consistent. Immediately before training the GNN, the protein-binding pocket and ligand graphs were concatenated to form a single input. Graphs were also switched from NetworkX objects to PyTorch Geometric objects. In total, our model processes 14 node features, 6 edge features, and 70 graph-level features.

### 2.4. Hyperparameter Tuning

To optimize our model, we performed hyperparameter tuning using the Tree-Structured Parzen Estimator (TPE) from the Hyperopt Python library. TPE sequentially chooses hyperparameters based on the achieved performance metrics from previously successful runs. The predetermined search space for the GNN-Hyperopt implementation included several different optimizers, loss functions, learning rates, numbers of hidden channels, and numbers of hidden layers (Table 3).

Our primary metric for evaluating model performance to determine ideal hyperparameters was the Pearson correlation coefficient (PCC). The hyperparameters that consistently produced the highest PCC were chosen for the experiment. Hyperopt directed us to use the adaptive movement estimation (Adam) optimization function; mean squared error (MSE) loss function; rectified linear unit (ReLU) activation function; and specific values for the number of hidden layers, nodes per layer, batch size, number of epochs, dropout rate, and learning rate. Hyperopt served an important role in optimizing the foundation of our model framework before we shifted to more involved and advanced techniques. As the model matured in the complexity of architecture and feature representation, Hyperopt was run multiple times to ensure we harnessed its highest capabilities during developmental stages. Hyperopt explored 30 different combinations of hyperparameters before choosing the optimal set.

### 2.5. Graph Neural Network Models

We developed a hybridized GNN model by combining three machine learning algorithms: GNN, extreme gradient boosting (XGBoost), and support vector regression (SVR). The GNN architecture was created using the PyTorch Geometric library, an extension of PyTorch designed for graph-based deep learning methods. The layers were implemented using the “GeneralConv” operator, which is PyTorch Geometric’s constructor for a standard GNN architecture. GeneralConv considers only node, edge, and graph-level attributes. All graphs were converted to PyTorch geometric “data” objects before training and testing, allowing the GNN to distinguish between various types of features. StructureNet processed graphs in batches of 128 to balance model efficiency and generalizability. Graphs for the entire dataset were generated, and the protein and ligand graphs were concatenated together prior to training and testing.

**Figure 3:**
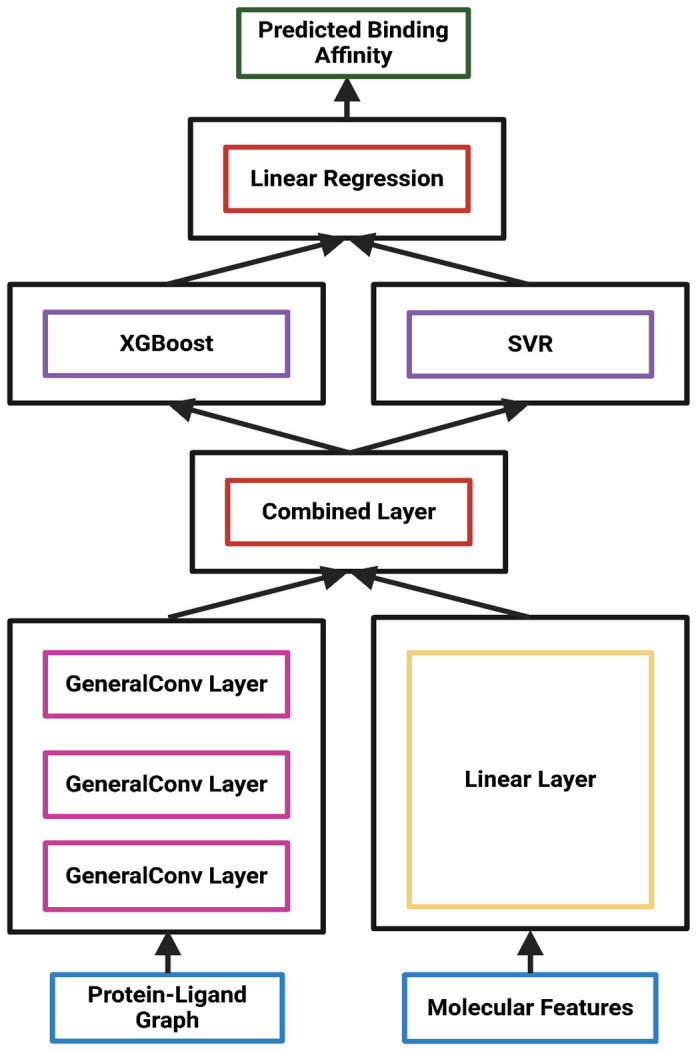
The training and testing framework for StructureNet. The protein–ligand graph and molecular features enter the GNN through GeneralConv and Linear layers, respectively. After GNN processing, they are combined and used to train XGBoost and SVM simultaneously. Predictions from SVM and XGBoost are generated, combined, and fed to linear regression to generate final predictions. All GNN layers used MAE as the loss function and Adam as the optimizer to update weights.

Data from the combined graphs initially followed two separate paths in the model architecture (Figure 3). The node and edge features were mapped to 128 hidden channels and passed through 4 hidden layers. Each hidden layer applied the rectified linear unit (ReLU) to introduce nonlinearity to the data. To prevent vanishing gradients, we implemented residual connections where each GeneralConv layer’s output was added to the subsequent layer. Meanwhile, the graph-level features were passed through one fully connected layer. The node and edge features from the combined protein–ligand graph were then concatenated with the outputs of the fully connected layers. After exiting a final dropout layer, the combined feature representation embeddings from the GNN were extracted and used as input for both the SVR and XGBoost algorithms. Hybridizing GNN with these two algorithms improves overall model performance by preventing overfitting and assessing feature importance. After SVR and XGBoost generated predictions, a linear regression model synthesized the predictions from the base learners. Outputted predictions from linear regression were then compared to their respective protein–ligand complex’s experimental binding affinity during training. This ensemble method improves model accuracy and provides an architecture capable of handling diverse binding affinity data.

### 2.6. Outlier Removal

We removed outliers from training and testing datasets by comparing model predictions with actual binding affinity values. Protein–ligand complexes with prediction errors exceeding three standard deviations (z-scores) were excluded from the dataset. Extreme binding affinity values can cause predictive inaccuracies and decreased model performance. We removed these outliers in an attempt to minimize bias in our results and boost the reliability of our model. On average, 30 complexes were removed for every 5000 in a dataset.

### 2.7. Integration of External Models

In line with our goal of investigating ideal feature representation, we developed a small subset of models that were hybridized with StructureNet. We built two standalone models that predicted binding affinity using either sequence or interaction-based descriptors. The sequence-based model employs a GNN to derive features from protein sequences and ligand SMILES strings. In contrast, the interaction-based model constructs a singular protein–ligand interaction graph for input to GNN, XGBoost, and RandomForest Regressor. The key differences between StructureNet and these two models are their emphasis on different categories of molecular descriptors (structural vs. interaction and sequence).

We hybridized StructureNet with the interaction-based model using two distinct approaches. In the first approach, the interaction graphs and structure graphs were passed through their respective GNNs. Instead of collecting the final predictions, we extracted the embeddings from both GNNs and combined them. The combined embeddings were then fed into a stacked regressor to generate binding affinity predictions. Our second approach used similar logic. Interaction graphs were fed through the interaction GNN while structure graphs were passed through the structural GNN. The output interaction data were fed through a stacked regressor while the structural data traveled through XGBoost and SVR. We then combined the data and used linear regression to make final predictions.

We followed a similar procedure to create merges with the sequence-based model. In our first approach, the structure graphs were passed through their GNN while the sequence graphs were generated. The sequence graphs were then combined with the structural GNN embeddings and passed through a stacked regressor to generate predictions. In our second approach, instead of concatenating embeddings to graphs and features, we directly stacked the predictions from the structure and sequence-based models to generate final predictions.

### 2.8. Ablation Study on Node Features

We conducted an ablation study on graph node features to assess individual descriptor performance. Nodes form the backbone for graph-based data representations, so appropriate node feature choice is important regarding overall model effectiveness [27]. Different features that contain similar information can introduce redundancy and inhibit model scoring power. Features unimpactful to accuracy impede model efficiency. Having insight into descriptors that drive prediction accuracy is critical for successful future work. We ran our model several times such that each node feature was removed in only one run and was the only node feature removed in that run. With 14 node features, we ran our model 14 times, omitting one node feature each time and returning it to its original place after testing. We used the PDBBind v2020 refined set to train and test StructureNet during the ablation study. Training and testing procedures did not change from previous methods. Except for input dimension (which was one less than baseline), all versions of the model had the same hyperparameters and architecture as the baseline model.

### 2.9. Molecular-Dynamics-Driven Conformer Ensembles for StructureNet

In our final analysis, we generated conformer ensembles for ten protein–ligand complexes to assess StructureNet performance on dynamical structural representations. We randomly selected ligand structural files used for molecular dynamics (MD) simulations from the PDBbind general and refined sets. Using these randomly selected ligand structures, PDB files were input into the ACPYPE server (https://bio2byte.be/acpype (accessed 27 April 2025)) to generate corresponding topology files (.itp and .pdb). The following parameters were selected during ligand preparation: the bcc charge model was applied, multiplicity was set to 1, the GAFF (General AMBER Force Field) atom type was used, and the net charge was set to auto. The output files, including the ligand .itp and .pdb files, were used in our MD simulations. MD simulations were performed using the full protein structure through the Visual Dynamics platform “https://visualdynamics.fiocruz.br/en-US/simulations (accessed on 1 April 2025)” [28]. Ligand files generated by ACPYPE were incorporated into the system setup. The force field parameters used for the simulation were AMBER03 for proteins and AMBER94 for nucleic acids. The SPC (simple point charge) water model was also selected. The AMBER force field was chosen because it offers well-parameterized torsion-angle potentials, a comprehensive and flexible set of atom types, accurate Lennard–Jones treatment of nonbonded interactions, and a full electrostatic model suited for crystalline environments—providing optimal specificity for our protein–ligand parameters [29]. The simulation box was defined as a cubic box with a box distance of 1.2 nm from the protein surface. The system was subjected to 5 ns of production simulation time under default production conditions provided by the Visual Dynamics platform. All MD simulations were conducted using the default GROMACS parameters. This methodology builds upon previously established molecular-dynamics-based screening approaches that use flexible ligand binding pocket conformations to increase virtual screening performance [30].

## 3. Results and Discussion

### 3.1. Overall Workflow of StructureNet

**Figure 4:**
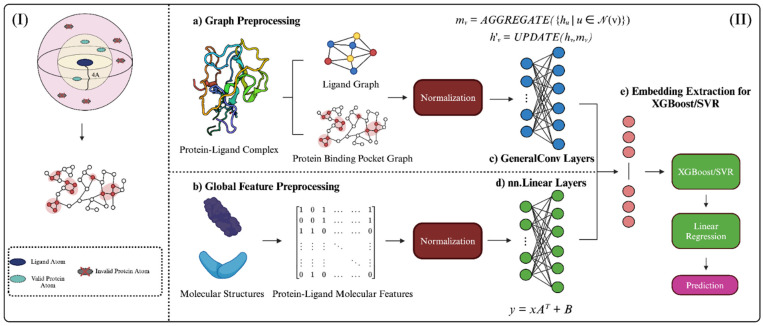
Graphical abstract depicting StructureNet workflow, from data preparation to binding affinity prediction. (**I**) Visual representation of the distance cutoff employed in protein binding pocket graphs. Pocket atoms >4 Å from the ligand were not considered. (**II**) The overall workflow of StructureNet in the broader context of other connected processes. (**a**) Each protein–ligand complex file from the respective PDBBind dataset was processed to generate a protein-binding pocket graph, ligand graph, (**b**) and protein–ligand molecular feature vector. Features were normalized using min–max normalization. Normalized graphs were inputted into the GNN (**c**,**d**) and concatenated to form a single input, after which embeddings were extracted and fed through SVR/XGBoost and linear regression to generate predictions (**e**).

We employed a structure-based graph neural network hybridized with XGBoost and SVR to predict the binding affinity of protein–ligand complexes (Figure 4). To begin graph preprocessing, datasets were imported for feature extraction. In our first phase of model evaluation, we imported the general and refined sets for protein–ligand binding complexes from PDBBind v2020. We also merged the general and refined sets separately to create a third dataset, the combined set for protein–ligand binding complexes, which was used in our second phase of model evaluation. Using combinations of RDKit functions and custom feature extraction functions, structural features for each protein–ligand binding complex were calculated. The protein binding pocket graph employed a distance cutoff (Figure 4), where protein atoms >5 Å from the ligand were not included in graph representation. Blank NetworkX graphs were created for each complex and populated with the respective node-, edge-, and graph-level features. All features were normalized with min–max normalization before training and testing. Similarly, global molecular features were calculated for each protein binding pocket and ligand molecule. These features were calculated and normalized separately, then stored in graphs as graph-level features.

During model evaluation, the graph-formatted data were input into StructureNet for training and testing (Figure 4). StructureNet uses convolutional layers to analyze structural data in atoms and bonds and fully connected linear layers to learn from global molecular features. Convolutional layers process features by aggregating information from neighboring nodes (AGGREGATE function). The aggregated information is then combined with existing node features to update and refine representation (UPDATE function). In fully connected layers, linear transformations are applied to the input feature vector (*x*) through multiplication with a weight matrix (*A^T^*) and adding a constant term to account for bias (*B*). All features are concatenated at the end of the GNN architecture, where embeddings are extracted. The embeddings from the GNN were then fed to trained XGBoost and SVR models. In line with our main goal of de novo drug discovery and high generalizability, we used XGBoost and SVR algorithms to boost model robustness and mitigate overfitting. Outputs from SVR and XGBoost are fed to a linear regression model to aggregate the stacked data and generate predictions. The final steps in model evaluation use data not included in the training phase of StructureNet, such as the DUDE-Z dataset or separate PDBBind datasets. We used K-fold cross validation (K = 8) when training and testing StructureNet to ensure that metrics represent predictions across the entire dataset. To evaluate model performance, we used Pearson correlation coefficient (PCC), area under ROC curve (AUC), mean squared error (MSE), mean absolute error (MAE), and R^2^. The following results detail the predictive performance of StructureNet, providing evidence that our structure-based approach to binding affinity holds relevance in future binding affinity prediction studies. StructureNet was developed using Python (version 3.11.8) on a system equipped with Apple M1 CPU and 16 GB RAM.

### 3.2. Model Evaluations Based on PDBBind v2020 Refined and General Sets

With the training workflow described (Figure 4), we began to evaluate StructureNet. The first phase of training and testing StructureNet used the refined and general sets from v2020 of PDBBind’s documentation. As discussed earlier, we performed eightfold cross validation and used z-score outlier removal for model evaluation on the refined and general sets. On the refined set, StructureNet achieved a 0.683 PCC, which indicates a moderate level of predictive power (Table 4 and Table S1). An AUC of 0.750 across the eight folds demonstrates StructureNet robustness to overfitting and its ability to capture meaningful trends in given feature spaces. Respective values for MSE and MAE of 1.964 and 1.125 show that there was a relatively small difference between the predicted and actual binding affinity values. An R^2^ of 0.466 reinforced the quality of model predictions and StructureNet ability to explain a moderate portion of dependent variable variance. Across the general set, StructureNet achieved an average PCC of 0.620, average of AUC 0.733, average MSE and MAE of 1.959 and 1.104, and an average R^2^ of 0.385 (Table 5 and Table S1). The metrics from general set testing showed a decrease in predictive performance when compared to the results from the refined set. In addition, StructureNet achieved higher metrics on the refined set data than it did with the general set data. This is likely because the general set is intentionally a more diverse dataset with a greater variety of protein–ligand complex structures. The general set (*n* = 14,127) is almost triple the size of the refined set (*n* = 5316) and includes 8811 binding complexes that do not meet the data quality specifications of the refined set. Additionally, the range of binding affinity values in the general set complexes span mM to fM, while the refined set values only span mM to nM. This increased diversity introduces a broader range of patterns that the model may not recognize as effectively as with the simpler refined set, which would explain the drop in performance.

### 3.3. Model Evaluation Based on Combined Set

To further test model performance, we merged the v2020 PDBBind refined and general sets to create a combined dataset with diverse protein–ligand complex representations. Specifically, we wanted to verify that the difference in StructureNet performance between the refined and general sets was due to increased diversity of molecular representation and not external factors. On the combined dataset, StructureNet achieved an average PCC of 0.645, average AUC of 0.738, average MSE and MAE of 1.956 and 1.107, and an average R^2^ of 0.416 (Table 6 and Table S1). The trend in metrics for the combined set reflects the trend in molecular diversity characterized by the combination. The metrics suggest higher predictive performance than the general set, but weaker than the refined set. The combined set contains both high-quality, uniform refined set complexes and the diverse general set complexes. StructureNet likely benefits from the inclusion of the refined set and its more predictable patterns but is weighed down by the increased complexity of the general set, which would explain its predictive performance being between that of the general and refined sets. This verifies our initial hypothesis on the described difference in predictive performance.

### 3.4. External Validation on Subsets of the PDBBind v2020 Dataset

After training and testing StructureNet on the refined, general, and combined sets, we moved onto our first phase of external validation. We conducted external validation to determine whether StructureNet can generalize well to unseen data. In our first phase of external validation, we experimented with several combinations of training and testing StructureNet using the general, refined, combined, and core sets. StructureNet_1_ corresponds to our model trained on the general set and tested on the refined set. Similarly, StructureNet_2_ corresponds to the model trained on the refined set and tested on the core set. Finally, StructureNet_3_ represents the model trained on the combined set and tested on the core set (Table 7).

Looking at the metrics, StructureNet showed high robustness but struggled to make accurate predictions in some of the external validation scenarios. StructureNet_2_ displayed high predictive power and robustness (Table 7). StructureNet was able to make high-quality predictions likely because the refined set (training data) and core set (test data) are similar in their high-resolution protein–ligand complexes. On the other hand, StructureNet_1_ was trained and tested on two datasets that were very different in their resolution and diversity. This model was trained on the general set, which emphasizes a diverse representation of protein–ligand complexes that have more irregular structures. The patterns our model learned from training did not apply when testing with less diverse binding affinity data in the refined set. Testing on the refined set likely contributed to a weaker ability to apply patterns learned during training. In StructureNet_3_, the model was trained on the diverse and large combined set. StructureNet_3_ achieved a PCC of 0.724 (Table 7). Though this indicates adequate model accuracy, predictions were not as accurate as with StructureNet_1_. This could be due to the fact that StructureNet_1_ was trained exclusively on complexes similar to those in the test set, whereas StructureNet_2_ was trained mostly on dissimilar ones. Although the training set for StructureNet_2_ contained the refined set (*n* = 5316), the representation of similar complexes was weakened by the much larger general set (*n* = 14,127). It is likely that not all of the patterns the model learned during training were applicable to test complexes. This would explain the minor drop in performance.

Evidently, StructureNet benefited greatly from training on protein–ligand complexes similar to those in the test set. The model performed well when complexes in both the training set and test set were of high quality (Table 7). However, a primary determinant of a binding complex quality is its structural resolution. Binding complexes with higher structural resolutions were assigned to the refined set, with only the highest-quality ones earning a spot in the core set. Therefore, it is reasonable to say that StructureNet generates accurate binding affinity predictions when train/test complex structures are similar. This is important because such a strength can be leveraged during future de novo testing. If training datasets are populated with complexes structurally similar to the de novo test complexes, predictions will likely be accurate. Though not necessarily new information, the link between structural similarity and functional similarity could be applied to training and testing StructureNet to achieve higher accuracy and robustness.

### 3.5. Model Evaluation on DUDE-Z Dataset

**Figure 5:**
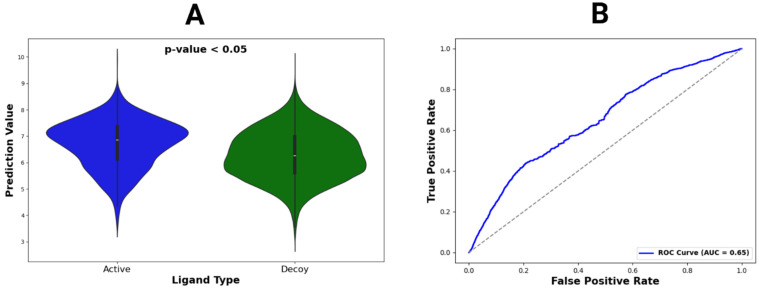
Violin plot and ROC curve for DUDE-Z dataset predictions. (**A**) The violin plot constructed for active vs. decoy ligands across all DUDE-Z complexes. Locations with wider color densities indicate higher frequencies of predictions. A *p*-value was calculated from *t*-tests comparing mean binding affinity predictions between active and decoy ligands. (**B**) The ROC curve, representing all predictions, with its AUC value given in the legend.

We tested StructureNet on the DUDE-Z dataset to further validate its performance by determining if it could distinguish between active and decoy ligands. Validating StructureNet with this dataset adds additional layers of complexity to the predictive task and tests how the model can differentiate true binding ligands from those that only appear similar. Therefore, the goal of this phase of testing was to determine whether our model could reliably distinguish true binders (active ligands) and non-binders (decoy ligands). For each protein, we constructed a violin plot to compare the prediction distributions between active and decoy ligands. In addition, we wanted to quantify StructureNet’s ability to distinguish between the two groups. The plot below represents predictions for the entire DUDE-Z dataset (Figure 5, Table S2). The median mark of the violin plots on the left shows StructureNet’s ability to separate high binders (active ligands) from low binders (decoy ligands) (Figure S1). The *p*-value calculated from a *t*-test of active ligand predictions versus decoy ligand predictions suggests that there is a statistically significant difference between the predictions between the two ligand types.

The success of StructureNet on the DUDE-Z dataset shows its capacity to distinguish these two classes of ligands through entirely structural descriptors. Also included is the receiver operating characteristic (ROC) curve for StructureNet predictions on the DUDE-Z dataset (Figure 5). The ROC curve compares a model’s true positive rate to its false positive rate. In this case, a binding affinity of 6 log units or 1 µM is the cutoff value between true active and true decoy. Any value at or above 6 log units is in the range for a true positive or false negative, while any value below 6 log units is either a true negative or false positive. We used the true positive rate (sensitivity) and false positive rate (1-sensitivity) to graph the ROC curve and find value for the area under the curve (AUC). An AUC value of 0.5 represents perfectly random predictions, while values closer to 1 indicate high discriminatory power. StructureNet achieved an AUC of 0.65 on the DUDE-Z dataset, which indicates moderate predictive ability and classification power (Figure 5 and Figure S2).

### 3.6. Benchmarking of StructureNet Against Similar Models

While the overwhelming majority of BAPDL models use sequence or interaction features to some extent, purely structure-driven approaches do exist. To our knowledge, the deep learning model used in this study is one of two existing BAPDL models that use entirely structural descriptors and traditional GNN architecture. Below, we compare the predictive performance of StructureNet and the other existing structure-based BAPDL model, named MPNN(PL) [31] (Table 8). Like StructureNet, MPNN(PL) uses a combined protein and ligand graph and employs a distance cutoff (though 1 Å smaller than the StructureNet distance cutoff). MPNN(PL) also uses differentiable node aggregation and update functions for data propagation, just like StructureNet. However, it is important to note that StructureNet uniquely calculates global molecular features for protein and ligand structures. To provide more insight into the predictive power of StructureNet, we compared our model with other successful BAPDL models encompassing other feature types below.

The most commonly reported metrics were MSE, MAE, and PCC. As shown above, StructureNet achieved a PCC of 0.683, an MSE of 1.964, and an MAE of 1.125 (Table 9). Apart from MPNN(PL) (Table 8), StructureNet achieved a higher PCC and a lower MAE than two models encompassing both structural and interaction features, DeepBindRG and GraphBAR [31,32]. DeepBindRG employs a CNN, which may limit the usefulness of structural data when compared to graph-based data representations in GNNs. GraphBAR uses a GNN similar to StructureNet but adopts much simpler features for model training and testing. The more improved structural representation of StructureNet (compared to GraphBAR) and graph-based learning process (compared to DeepBindRG) explains its higher predictive power. Interestingly, however, GraphBAR produced a lower MSE than StructureNet and DeepBindRG. StructureNet outperformed LigityScore on the v2016 core set but was outperformed on the v2013 core set. However, we achieved lower MSE/MAE values than both iterations of LigityScore testing (Table 9) [33]. LigityScore uses a CNN architecture and heavily relies on interaction and sequence descriptors. The LigityScore evaluation methodology was focused primarily on evaluating scoring power and did not expose the model to any true external validation scenarios. The datasets used for model evaluation, the CASF-2013 and CASF-2016 scoring power benchmarks, are simply subsets of the PDBBind general and refined sets. This approach is well-suited for theoretical benchmarking but lacks the real-world challenges and complexities in practical drug discovery workflows. Therefore, it cannot be clearly said that LigityScore shows higher generalizability and pattern recognition capabilities than StructureNet. StructureNet was fully outperformed by HNN-denovo, SIGN, and Pafnucy [5,33,34,35]. HNN-denovo is a combination of several models, and uses structural, interaction, and sequence descriptors to make highly accurate predictions [5]. SIGN employs polar coordinate-inspired attention layers to model spatial relationships and pairwise interactive pooling to simultaneously capture intermolecular interactions. It makes more accurate predictions than StructureNet primarily through enhanced spatial representation and dynamic feature representation [34]. Pafnucy uses a CNN architecture and discretizes protein–ligand complexes into 1 Å grids for an enhanced 3D structural representation [35]. SIGN and Pafnucy, both of which have higher predictive power than StructureNet, offer more polished structural molecular representation [34,35]. DeepBindRG and GraphBAR, which were outperformed by our model, placed less of an emphasis on structural molecular representation [31,32]. Though not an explicit proof, the correlation between structural descriptor emphasis and model accuracy and generalizability offers interesting insights into the importance of structural, interaction, and sequence descriptors.

While other structure- and GNN-based BAPDL models exist, most incorporate non-traditional or external deep learning techniques to improve model accuracy (Table 9).

### 3.7. Inclusion of Interaction/Sequence Feature Frameworks

As portrayed above, models that include sequence and interaction descriptors generally output more accurate predictions on train/test data than their structure-exclusive counterparts (Table 8 and Table 9). However, as described earlier, many models that use these features often resort to memorizing data patterns and tend to generalize poorly to unseen data. Though they are more powerful on paper, these models fail when predicting data for unseen complexes. To explore this limitation further, we integrated StructureNet with two specialized DL frameworks designed to emphasize protein–ligand descriptors not included in StructureNet. One of the frameworks focuses on sequence features such as protein sequences and ligand SMILES strings, while the other model focuses on protein–ligand interactions. Importantly, both standalone models which we hybridized with exhibit high predictive power on both training/testing and external validation. This ensures that our hybridized models are learning how to make binding affinity predictions from interaction- and sequence-based features without simply memorizing patterns specific to the data used.

The two additional models we integrated with are specialized in extracting sequence-based and interaction-based features, as opposed to structural features. The sequence model derives features from the protein pocket’s amino acid sequence and the ligand’s SMILES format to process them as graphs in an XGBoost + RF stacked regressor. The interaction-based model generates a graph of the protein–ligand complex with interaction features embedded and uses both GNN and a hybrid XGBoost + RF regressor to generate predictions. Here, we detail the predictive power of two possible combinations of architecture trained and tested on subsets of the PDBBind v2020 Refined Set.

**Figure 6:**
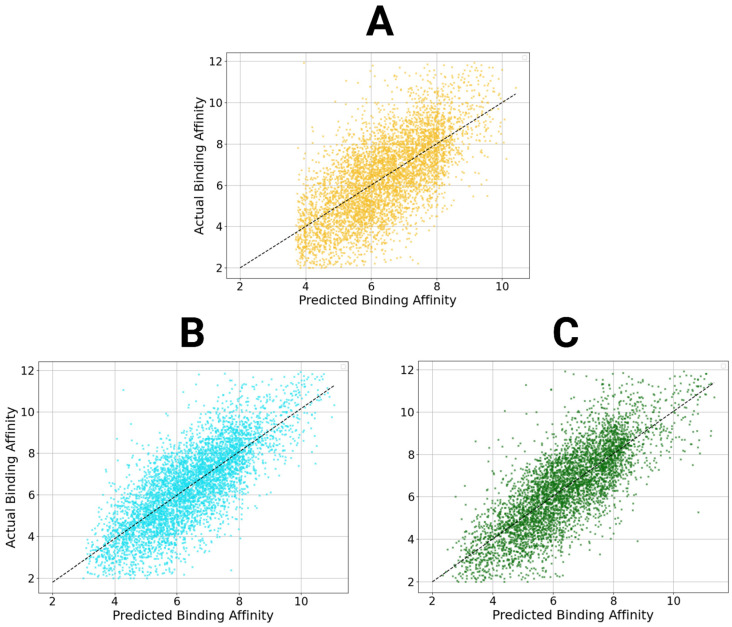
Scatter plots of StructureNet and hybridized model predictions. Scatter plots of the predictions from StructureNet (**A**), Structure + Sequence V2 (STR + SEQ, (**B**)), and Structure + Interaction V2 (STR + INT, (**C**)) against actual binding affinity values.

Both Structural + Interaction V1 and V2 displayed significantly higher predictive power than the standalone StructureNet model (Table 10, Figure 6). The inclusion of interaction features enhanced the model’s ability to capture more nuanced protein–ligand relationships that structural features alone could not fully describe. The higher predictive performance shown in the metrics suggest that the hybrid model was better able to account for complex interactions and dependencies, which led to improved generalization and accuracy. Similarly, Structural + Sequence V1 and V2 showed much higher accuracy and performance than StructureNet did as a standalone model. The two categories of features included in the merges complemented each other by capturing different aspects of the protein–ligand interactions. The combination succeeded in capturing spatial information and residue-level properties simultaneously, contributing to its strong predictive performance. Notably, both hybridized models were better at predicting the affinities of low binders than StructureNet as a standalone model.

After training and testing each hybridized model, we externally validated StructureNet and the best of the two hybridized models, Structural + Interaction V2 (STR + INT) and Structural + Sequence V2 (STR + SEQ), on a small group of well-known drugs: sulindac, sunitinib, and mebendazole. We downloaded the 3D crystal structures of the drugs complexed with their native proteins from the RCSB website and sourced the drugs’ experimental binding affinities from the BindingDB database. All three models were trained on the previously used combined set and tested on the subset of drugs described above. The protein–ligand-binding complexes 3g0f, 4agd, 4ks8, 4qmz, 6jok, 6nfz, and 6ng0 correspond to the drug sunitinib, while 2kaw, 3rx3, 3u2c, and 4wev contained sulindac. Complex 7odn was the sole complex for mebendazole.

**Figure 7:**
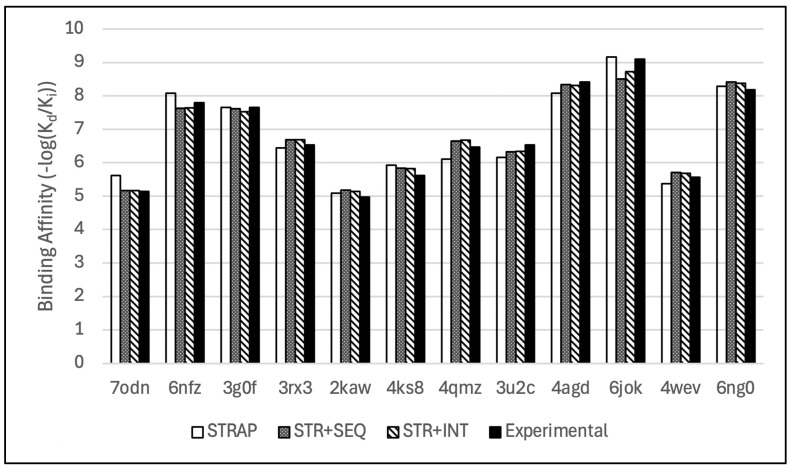
Bar chart comparison of de novo binding affinity predictions from the hybridized models with their respective experimental values.

The PCC for the predictions from StructureNet, STR + SEQ, and STR + INT were 0.96, 0.97, and 0.97, respectively (Figure 7, Table S3). STR + SEQ and STR + INT displayed higher predictive power than StructureNet as a standalone model, but the difference was marginal. From the train/test split, the difference in PCC between StructureNet and STR + SEQ/STR + INT was large (0.077/0.090, respectively). However, this only translated to a PCC difference of 0.01 in validation testing. Though both hybridized models were much more powerful on the refined set, their superiority to StructureNet when tested on unseen data was insignificant. This phase of testing further emphasizes that sequence and interaction descriptors do not contribute significantly to model generalizability in scenarios outside of standard training and testing. It is evident that both hybridized models were unable to apply the insights from additional descriptors to these unfamiliar drug complexes. While the models performed well during normal training and testing phases, our model’s main goal is to be used for rapid virtual screening and de novo drug predictions. Our external validation testing with mebendazole, sulindac, and sunitinib show that StructureNet has high potential for applications in de novo testing for drug discovery. Future work will focus on improving standalone models like StructureNet by further exploring unique and insightful structural descriptors.

### 3.8. Evaluation of StructureNet Predictions Against AutoDock Vina

As a final benchmark, we compared the performance of StructureNet predictions to binding affinity predictions obtained from AutoDock Vina. We used 12 protein–ligand complexes that were virtually docked by AutoDock Vina to generate predictions from StructureNet, STR + SEQ, STR + INT, and STR + SEQ + INT. We randomly selected 12 receptor–ligand-binding complexes from a large pool of endogenous ligand complexes that we had manually docked for use in external studies. Vina uses a custom scoring function to predict binding free energy values after performing virtual protein–ligand docking simulations [36]. We converted Vina binding free energy into binding affinity using the Gibbs free energy equation:

$$K_{d}=e^{∆G/RT}$$

where *K_d_* is the predicted binding affinity in molar, ∆*G* is the Gibbs free energy prediction from Vina in kcal/mol, *R* is the gas constant in units of kcal/mol, and *T* is the temperature at standard state (298 K).

**Figure 8:**
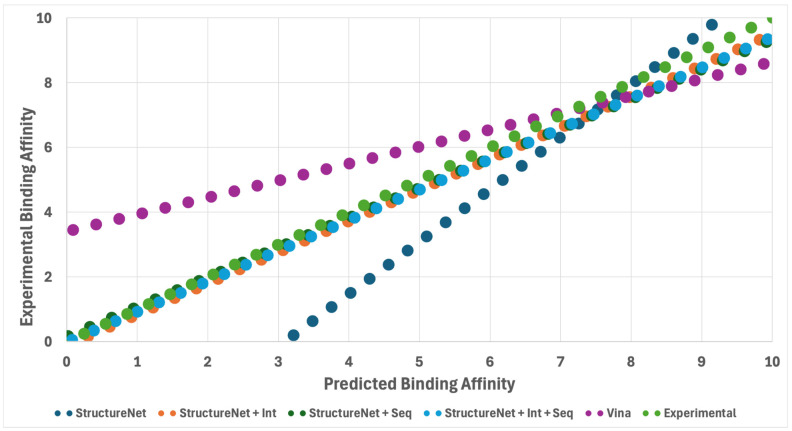
Trendlines showing the relationship between model predictions (StructureNet, StructureNet + Int, StructureNet + Seq, StructureNet + Seq + Int, AutoDock Vina) and experimental binding affinity values. Twelve docked complexes were used to generate binding affinity values, which were converted to −log(*K_d_*) for smoothness of fit. The purple dotted line is a reference line representing the experimental binding affinity values and a perfect linear correlation.

The PCCs for StructureNet, StructureNet + Int, StructureNet + Seq, StructureNet + Int + Seq, and Vina were 0.70, 0.76, 0.72, 0,73, and 0.64, respectively (Figure 8). StructureNet and all hybridizations outperformed Vina in predicting binding affinity values over the twelve docked complexes. Interestingly, the trendline for StructureNet is inverted with respect to the trendline for Vina. Vina’s trendline has a lower slope, while the trendline for StructureNet has a higher slope. This suggests that, although StructureNet outperformed Vina in predictive accuracy, they generated predictions on opposite scales. While StructureNet is “ranking” complexes more accurately than Vina, it is doing so on a reversed numeric scale. One framework is outputting higher predictions while the other is inputting lower ones. Regardless, StructureNet and all of its hybridizations showed higher predictive power than AutoDock Vina predictions from virtual docking.

### 3.9. Ablation Study on Structure-Based Descriptors

StructureNet generates predictions by learning from a large and diverse set of structural molecular descriptors. We performed an ablation study on node (atom) features to provide insight into which features drive model predictions. Furthermore, we wanted to examine the extent to which Voronoi and spherical harmonics contributed to model accuracy. If our custom features impacted accuracy more than the built-in RDKit/Biopython descriptors, it would suggest they are more physicochemically informative and useful to the StructureNet algorithm. One-by-one, each node feature was omitted, and the model was tested three times on the refined set. The feature being evaluated was then added back before the removal of the next feature. Metrics were averaged over three runs of training and testing.

**Figure 9:**
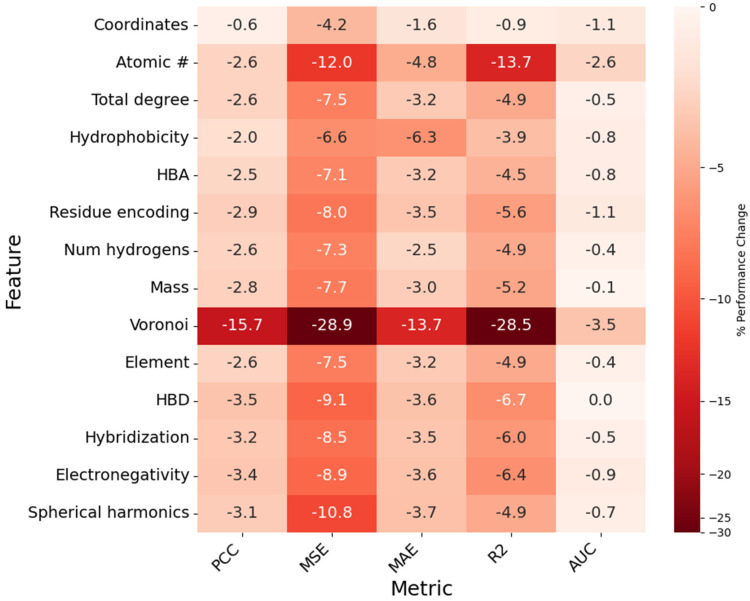
Heatmap showing the impact of node feature removal on model PCC, MSE, MAE, R^2^, and AUC. Each cell represents the percent difference from baseline value for a given metric (column) when the corresponding feature (row) is removed. Cells are assigned a value, as a percentage (%), and a color, which is determined by the magnitude of the percent difference.

Evidently, most node features resulted in a consistent and minor decrease in model performance when they were removed (Figure 9, Table S4). Removing atomic coordinates did not cause significant changes in any metric. Removing the atomic number feature caused the second most impactful increase for MSE/MAE (+0.235/0.054) and decrease for R^2^/AUC (−0.064/0.019). Removing hydrophobicity, HBA status, and total degree did not significantly impact any metric, suggesting they provide redundant information. The spherical harmonics feature was responsible for the third greatest increase in MSE (+0.213), fourth greatest increase in MAE (0.042), and fifth greatest decrease in PCC (−0.021) when removed. The element name, HBD, hybridization, and atomic mass descriptors were responsible for a low to moderate impact on most metrics, except for PCC (HBD, −0.024), R^2^ (HBD, −0.031), and AUC (hybridization, −0.004). The removal of spherical harmonics descriptors hurt some metrics (PCC, MSE) more than others (MAE, R^2^, AUC).

Interestingly, removing Voronoi caused the most significant decrease in model performance across all metrics. Model PCC, R^2^, and AUC decreased by 15.7%, 28.5%, and 3.5%, while increasing MSE and MAE by 28.9% and 13.7%, respectively. This is notable because binding affinity is controlled by biochemical interactions, yet Voronoi tessellation, the most impactful node feature, represents purely geometric calculations. This suggests that along with basic biochemical data, additional molecular descriptors are not inherently more impactful than their purely geometric counterparts. Even though geometric features like Voronoi do not explicitly describe molecular characteristics, they detail spatial relationships and atomic arrangements which often capture interaction patterns indirectly. Previous DL approaches to binding affinity prediction that lean heavily on global molecular descriptors would likely experience improvements from including similar geometric information.

### 3.10. Molecular-Dynamics-Derived Ensembles Improve the Prediction Accuracy of StructureNet

The ablation study features Voronoi tessellation as the primary contributor to StructureNet’s predictive accuracy (Figure 9). While Voronoi does not explicitly capture biophysical molecular properties, it encodes local geometric features. These features strongly shape StructureNet’s spatial perception. As a result, the Voronoi representation of a given binding complex is sensitive to variations in binding conformations, since these can significantly alter surface geometry. However, until now, our model has only been trained with static structures showing a single binding conformation. To determine if StructureNet’s prediction accuracy improves with different binding conformations, we ran MD simulations on ten protein–ligand complexes chosen at random from the combined set [27]. StructureNet integrates graph-based structural learning with MD-generated structures to capture protein–ligand conformational flexibility. It uses MD-generated ensembles of protein–ligand complexes to account for binding site dynamics. We trained it on multiple conformations from MD runs and combined this with structural learning to better model real-world binding behavior.

**Figure 10:**
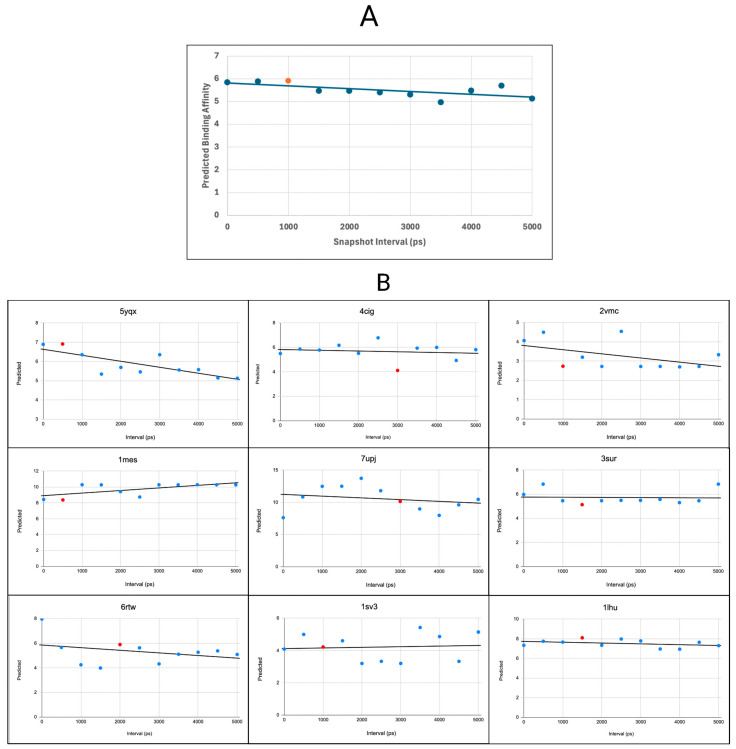
(**A**) Scatter plot with trendline showing the distribution of the exemplary conformer’s binding affinity predictions across the duration of its MD simulation. The data point representing the most accurate prediction is given above in orange. (**B**) Scatter plots for all other conformer ensembles in the dataset. The data points representing the most accurate predictions are given above in red.

Each simulation lasted 5 ns, and conformer ensembles were collected for each binding complex every 500 ps. These ensembles represent a range of different structural conformations for the binding complex. We then trained StructureNet on the combined dataset, excluding the ten selected complexes, and evaluated its performance on both the ten original complexes and all conformers generated. For each complex, we compared the prediction from the original complex to the predicted affinities for the generated conformers. The table and line graph below show an example of prediction distributions across the conformers for the protein–ligand complex 4g8n, along with the original prediction and experimental binding affinity (Table 11, Figure 10).

Binding affinity predictions fluctuated across individual complexes and their conformers (Figure 10, Table S6). Although they represent the same protein–ligand complex, each conformer within the ensemble captures different structural configurations of the protein binding pocket and ligand. These changes influence the binding affinity between the two molecules and explain the fluctuations in predicted binding affinity across time intervals. For nearly every binding complex, at least one conformer led to a more accurate binding affinity prediction by StructureNet than the original prediction based on the reference structure (Table S6). The majority of conformer predictions that outperformed their corresponding reference structure predictions emerged during the early stages of the MD simulations. The ensembles showing the most optimal binding affinity predictions indicate the most stable conformational states within our MD simulations. These structures often correspond to energy minima on the free energy landscape, where the ligand and receptor show geometries that maximize favorable interactions such as hydrogen bonding, hydrophobic contacts, and favorable electrostatic interactions. As a result, they show representative configurations of thermodynamically preferred binding poses, showing the structural aspects of complex stability [37]. Two complexes achieved their most stable structure within 500 ps, while six others reached this state in the first 2000 ps (Table S6 and Table 11). The remaining two complexes achieved their most stable structural configuration within 3500 ps. In these early stages, the binding complex optimizes many of its interactions (such as hydrogen bonds and Van der Waals contacts), which relaxes the molecular structures and contributes to a more optimal binding pose [38]. This stage of the MD simulation resembles the binding complex transitioning into a more enthalpically favorable state, which is closer to the native binding conformation than the original crystal structure [39]. In fact, extremely short MD simulations (~1.2 ns) contain enough molecular information for true binders and false positives to be reliably distinguished from one another [40]. This strengthens the claim that early stages of MD simulations provide insight into in vivo protein–ligand binding outcomes.

To verify our protocol, we compared the predicted binding affinities to the number of protein–ligand interactions observed in each complex’s reference structure and its corresponding conformers. Since binding affinity quantifies the potency of a protein–ligand interaction, higher binding affinity values should be reflected by a greater number (or strength) of intermolecular interactions. We characterized the differences in protein–ligand interactions using the BINANA algorithm and compared the number of computed interactions to the binding affinity predicted by StructureNet. We selected one conformer at random to compare to each reference structure [41].

**Figure 11:**
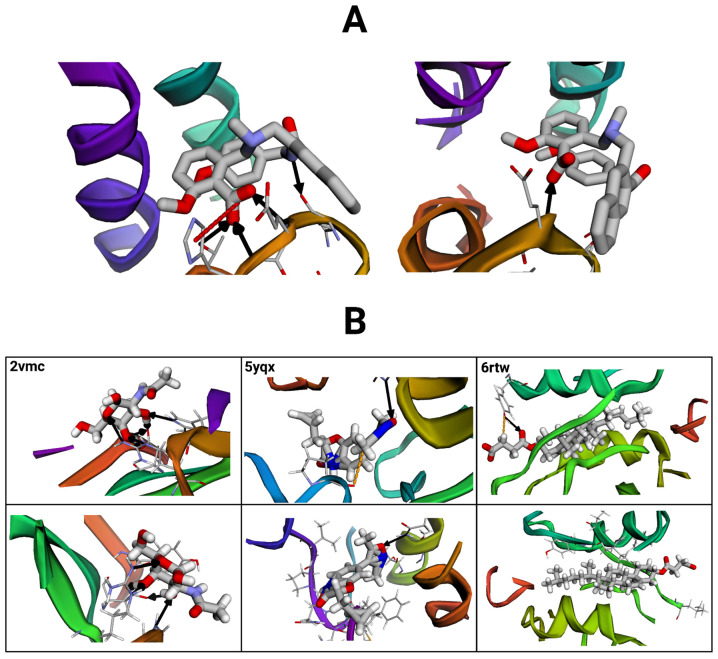
Protein–ligand complex interactions visualized with the BINANA (version 2.2) online software. Black arrows represent hydrogen bonds, red dashed lines depict salt bridges, and orange dashed lines represent metal contacts. (**A**) Visualization of the exemplary reference structure (left) compared to a selected conformer. Molecular files were dehydrogenated and maximized for clarity. (**B**) Visualizations of three additional reference structure–conformer pairs (paired vertically). Images on the top represent reference structures, and those below represent their conformer pairs.

For every conformer–reference structure pair that was selected, differences in StructureNet prediction values were directly reflected in the number of intermolecular interactions (Table 12 and Table S5, Figure 11 and Figure S3). Figure 11 and Table 12 show that StructureNet predicted a binding affinity of 5.496 log units for the reference structure and 4.929 log units for the randomly selected conformer. The reference structure, which was assigned to have a greater binding affinity, contained more intermolecular interactions than the selected conformer with a lower predicted binding affinity (Table 12, Figure 11). The consistency of this pattern throughout our selected conformers validates StructureNet’s learning rationale and its ability to connect higher intermolecular interaction activities with stronger binding affinities. These results also verify that the MD simulations were correctly carried out, and protein–ligand interaction strengths varied throughout the simulations in the expected manner.

However, conformers with a higher predicted binding affinity than the experimental value do not necessarily represent more stable binding structures because binding stability depends on both binding free energy (∆G), conformational entropy, and kinetic accessibility. A conformer with a stronger affinity than the experimentally determined binding affinity is almost always an artifact of the predictive model at play [42,43]. These models often focus too much on certain types of interactions, such as how strongly atoms attract each other, and ignoring other important factors that affect binding. As a result, they tend to give high scores to conformations that seem good in theory but do not actually occur in cellular conditions. Some of these predicted shapes might sit in deep energy wells, but do not bind well in solution because of poor on-rates and off-rates. This makes them less thermodynamically favorable compared to all possible conformers. The most stable binding conformation is represented by the experimental binding affinity [43].

## 4. Limitations

While StructureNet is powerful in binding affinity prediction, it carries several limitations. Primarily, the model’s deep learning framework relies upon complex algorithms and input types to generate predictions. The model synthesizes a complex custom GNN with three separate machine learning models to boost model robustness and generalizability. Graphs need to be individually crafted and require complicated molecular and geometric feature calculations. The high computational demand from input type complexity and model architecture results in high training times, especially when using larger datasets like the general and combined sets. In model optimization, improvements in efficiency and runtime often result in higher model accuracy and predictive power [44]. StructureNet could benefit from direct runtime optimizations to improve both ease of use and prediction accuracy.

Another critical limitation is that StructureNet’s core approach assumes static structural features. Drug–target complexes are dynamic by nature, and changes in structure often play key roles in binding. Proteins often undergo induced fitting mechanisms, making important structural changes to fit a specific ligand. Static models that ignore conformational changes in protein structure may miss key binding events [45,46]. Integrating flexibility patterns into the prediction workflow could improve accuracy in cases where protein flexibility heavily influences binding, such as with flexible enzymes and allosteric inhibitors [47]. Evidently, including dynamic representations of protein–ligand binding complexes has potential to improve upon prediction accuracy when compared to using the original reference structure (Figure 10, Figure 11 and Figure S3, Table 12, Tables S5 and S6). Neglecting flexibility in our traditional train–test split and approved drug testing experiments represents another important limitation that may be hurting StructureNet predictive power [48].

The process of data removal also introduces potential biases into the dataset. In this study, we used z-score outlier removal in which data points with z-scores exceeding +3/−3 were removed from the dataset. This process was designed to eliminate extreme values in the dataset that would skew StructureNet predictions and reduce predictive power. However, this process could exclude protein–ligand complexes that represent meaningful variations. Since StructureNet was designed to be applicable to de novo testing, it must learn from a diverse training set. Omitting the highest and lowest binders could have negatively impacted StructureNet’s ability to generalize and apply patterns learned from training data to de novo complexes. Similarly, when training and testing with the general and combined sets, we removed all complexes that reported binding affinity values using IC50 units. These complexes were unable to be included because IC50 is measured on a separate scale than *K_d_* and *K_i_*. Conversions between the two units depend on specific experimental conditions which are unavailable for the PDBBind datasets we used. Since these complexes represent additional diversity, excluding them from model workflow may limit model generalizability and introduce distribution bias into the datasets.

## 5. Future Directions

Results from StructureNet indicate that structural molecular data provide sufficient description for binding affinity predictions. In addition, the pattern recognition issues that arise from sequence and interaction descriptors can be effectively mitigated by merging specialized standalone models. Future work will continue to develop StructureNet and successful merges in search of the ideal combination of descriptors. Although StructureNet as a standalone generates less accurate predictions on train/test data than the merges, it performs equally well on unseen data and is inherently more efficient since it is a less complex model. However, we cannot ignore that merged models were much more powerful with train/test data. Fleshing out each model’s full capabilities and assessing its strong and weak points will help us strike a balance between efficiency and predictive power.

Another important future direction relates to the high potential of geometric descriptors in binding affinity prediction. Voronoi tessellations were by far the most impactful node feature to model accuracy despite not explicitly describing any molecular characteristics. Spherical harmonics calculations were impactful to some metrics and less impactful to others. It is possible that including more mathematical descriptors that implicitly describe molecular characteristics would boost model accuracy and generalizability. For example, Delaunay Triangulation would provide complementary information to Voronoi diagrams and enhance the detectability of binding sites. Calculations such as alpha shapes describe molecular concavity and convexity, which play important roles in molecular interactions. Enhancing the topological descriptions of molecules in StructureNet graphs would likely contribute to enhanced predictive power [49].

Different constructors are also being explored to build the GNN architecture. GeneralConv was employed in this study, which constructs a simple GNN architecture with node, edge, and graph-level features. Graph attention networks (GATs) are another popular choice in BAPDL models since they can capture complex patterns sometimes invisible to other architecture types. This change would likely improve StructureNet predictive power on both train/test and external validation data. Graph isomorphism networks (GINs) use the Weisfeiler–Lehman (WL) test to determine graph isomorphism, which details the structural similarity between two graphs. GINs better retain detailed structural information and would help the model further understand similarities and differences between molecules. This adjustment would likely have the greatest impact on DUDE-Z testing, where differentiation between active and decoy molecules is the primary task.

Finally, we intend to explore the connection between structural similarity and functional similarity in the context of StructureNet predictions. External validation with the general, refined, and core sets revealed that StructureNet exhibits high predictive power when trained on complexes structurally similar to the test set complexes. We plan to expand testing on well-known drugs by creating custom training datasets that emphasize structural similarity with the test complexes. This further testing would provide more insight into whether or not our model’s predictive accuracy is consistently higher when considering this relationship. Analyses of similarity measures such as Tanimoto coefficients, chemical fingerprints, and shape-based similarity methods represent promising avenues for future work [50,51].

## 6. Conclusions

In this work, we developed and tested StructureNet, a hybridized deep learning model for predicting protein–ligand binding affinity. StructureNet exclusively uses structural molecular descriptors to learn patterns and generate predictions and aims to address the limitations introduced by sequence- and interaction-based descriptors. StructureNet was trained and tested on the PDBBind v2020 refined and general sets and externally validated on the PDBBind v2016 core set and the DUDE-Z scoring dataset. Our model outperforms the only existing structure-based GNN model, as well as several deep learning models that do include interaction and sequence features [22]. StructureNet achieved a PCC of 0.683 and AUC of 0.743 on the refined set, displaying its predictive power. External validation on the DUDE-Z dataset confirmed StructureNet’s ability to distinguish between active and decoy ligands. Benchmarking on a subset of well-known drugs further validated StructureNet potential in de novo applications for drug discovery. Testing StructureNet on conformers generated by MD simulations supported the notion that static protein–ligand complex representations may hinder model accuracy and generalizability. This physics-informed integration of MD simulated protein–ligand structural ensembles improved model performance by capturing realistic interaction patterns that are often missed in static representations.

The limitations described above outline promising avenues for future work. Given the model’s complex architecture and feature extraction process, there is room for improvement in efficiency. Optimizing StructureNet’s standalone and hybridized models represents the most promising route. Ablation study on node features highlighted the most descriptive features as well as ones that provide redundant information. Future versions of models will conduct ablation studies on edge and graph descriptors and remove redundant features to boost efficiency. Incorporating GAT or GIN architectures would also allow StructureNet to learn patterns in molecular structure with more nuance. The resulting model would likely handle outliers in a more calculated manner from a more detail-oriented learning process. Since outliers represent important molecular diversity, keeping them in training and testing datasets would further boost model reliability and learning. Future versions of StructureNet will also place more emphasis on dynamic representations of binding complexes. Incorporating MD simulation conformers more thoroughly in train–test datasets would provide the model with a broader and more applicable understanding of how molecular structure and intermolecular interactions are connected and how both characteristics influence binding affinity.

## Figures and Tables

**Table 1:** Node and edge features used to represent protein-binding pocket and ligand structures in graphs. Descriptors in nodes and edges detail atom- and bond-related properties, respectively.

Feature Name	Feature Description
Atomic number	The element’s atomic number.
Total degree	Total number of bonds formed with other atoms.
Hybridization	The atom’s hybridization state.
Total number of hydrogens	The total number of hydrogens bonded to the atom.
Mass	The atom’s atomic mass.
Hydrogen bond donor *	The atom’s hydrogen bond donor status.
Hydrogen bond acceptor *	The atom’s hydrogen bond acceptor status.
Hydrophobicity index *	The atom’s hydrophobicity.
Electronegativity *	The atom’s electronegativity.
Element name *	The element’s chemical abbreviation.
Residue encoding *	One-hot encoding of the residue type (amino acid for protein atoms, other for ligand atoms).
Voronoi regions *	Constructs a Voronoi diagram and calculates spatial relationships between atoms.
Spherical harmonics *	Calculates angular properties of the atomic coordinates in ℝ^3^.
Coordinate list *	The Cartesian coordinates of the atom in ℝ^3^ (x, y, z).
Bond type	The type of bond represented by an edge.
Conjugation status	Whether a bond is part of a conjugated system.
Ring status	Whether a bond is part of a cyclic ring structure.
Bond order *	The number of chemical bonds between two atoms.
Bond length *	The length of a bond between two atoms.
Electrostatic interactions *	The Coulombic attraction/repulsion between two charged atoms.

**Table 2:** Molecular features included in protein-binding pocket and ligand graphs. Unlike node (atom) and edge (bond) features, these molecular features represent global molecule-level characteristics and are stored as graph-level attributes.

Feature Name	Feature Description
Asphericity	Calculates molecular asphericity.
Eccentricity	Calculates molecular eccentricity.
exact molecular weight	Describes exact molecular weight.
CSP3	Describes the number of C atoms that are SP3 hybridized.
Inertial shape factor	Describes molecular inertial shape factor.
Number of aliphatic carbocycles	Calculates the number of aliphatic carbocycles for a molecule.
Number of amide bonds	Calculates the number of amide bonds within a molecule.
Number of aromatic carbocycles	Calculates the number of aromatic carbocycles for a molecule.
Hall–Kier alpha	Measures the degree of connectivity and branching within a molecule.
H-bond acceptors	Calculates the number of hydrogen bond acceptors for a molecule.
H-bond donors	Calculates the number of hydrogen bond donors for a molecule.
Heteroatoms	Calculates the number of non-carbon atoms in a molecule.
Rotatable bonds	Calculates the number of rotatable bonds in a molecule.
MolLogP *	Calculates the lipophilicity of a ligand (0 for protein).
NHOH count *	Calculates the number of amine and hydroxyl groups in a molecule.
NO count *	Calculates the number of nitrogen and oxygen atoms in a molecule.
QED *	Calculates the quantitative estimate of drug-likeness for a molecule.
PEOE_VSA1-14	Calculates the partial equalization of orbital electronegativities for a molecule.
SMR_VSA1-8	Measures the Van der Waals surface of a molecule categorized by ranges of molar refractivity.
SlogP_VSA1-12	Measures the Van der Waals surface of a molecule as divided into regions based on logP values.
VSA_EState1-10	Measures the Van der Waals surface of a molecule as divided into regions based on electrostatic state indices.
MolMR	Calculates the molar refractivity of a molecule.
TPSA	Returns a molecule’s TPSA value.
Kappa1	Calculates molecular Hall–Kier kappa1 value.
LabuteASA	Calculates Labute’s approximate surface area for the molecule.
PBF	Returns the plane of best fit (PBF) for a molecule.
Chi0n/v	Calculates the chi index of a molecule that characterizes its structural attributes.
Morgan fingerprint	Describes the surrounding environments/substructures of atoms in a molecular fingerprint.
Hashed torsion topological fingerprint	Describes the torsion angles in a molecular fingerprint.
Hashed atom pair fingerprint	Describes the shortest paths between bonded atoms in a molecular fingerprint.

**Table 3:** Search space of hyperparameters tested for the StructureNet model architecture. Hyperparameters chosen by Hyperopt and used for StructureNet are given in bold.

Hyperparameter	Search Space
Hidden channels	64, **128**, 256
Optimizer	**Adam**, Adagrad, RMSProp, Huber
Hidden layers	2, **3**, 4, 5
Learning rate	0.005, **0.001**, 0.05, 0.01
Loss function	MSE, **MAE**
Dropout rate	0.3, 0.4, **0.5**
Edge dropout rate	0.05, **0.1**, 0.5
Batch size	32, 64, **128**, 256
Epochs	50, 75, **100**

**Table 4:** Predictive power of StructureNet on the PDBBind v2020 refined set.

Fold	MSE	MAE	R^2^	PCC	AUC
Average	1.964	1.125	0.466	0.683	0.750
Fold 1	1.887	1.119	0.458	0.677	0.713
Fold 2	1.958	1.135	0.466	0.683	0.777
Fold 3	2.075	1.133	0.464	0.681	0.749
Fold 4	2.025	1.151	0.466	0.683	0.728
Fold 5	1.755	1.066	0.477	0.691	0.740
Fold 6	2.057	1.151	0.461	0.679	0.752
Fold 7	2.050	1.141	0.455	0.675	0.734
Fold 8	1.899	1.109	0.479	0.692	0.755

**Table 5:** StructureNet predictive power on the PDBBind v2020 general set.

Fold	MSE	MAE	R^2^	PCC	AUC
Average	1.959	1.104	0.385	0.620	0.733
Fold 1	1.960	1.116	0.388	0.623	0.726
Fold 2	1.862	1.073	0.398	0.631	0.747
Fold 3	1.983	1.113	0.386	0.621	0.727
Fold 4	1.975	1.103	0.404	0.635	0.745
Fold 5	1.957	1.102	0.382	0.618	0.726
Fold 6	2.066	1.142	0.361	0.601	0.711
Fold 7	1.873	1.074	0.383	0.619	0.737
Fold 8	1.995	1.114	0.375	0.612	0.749

**Table 6:** StructureNet predictive performance on the combined set.

Fold	MSE	MAE	R^2^	PCC	AUC
Average	1.956	1.107	0.416	0.645	0.738
Fold 1	1.981	1.107	0.422	0.650	0.739
Fold 2	1.959	1.117	0.402	0.634	0.724
Fold 3	1.986	1.113	0.437	0.661	0.746
Fold 4	1.928	1.109	0.422	0.635	0.745
Fold 5	1.912	1.098	0.419	0.647	0.741
Fold 6	1.995	1.120	0.415	0.644	0.731
Fold 7	1.906	1.087	0.431	0.656	0.763
Fold 8	1.995	1.115	0.380	0.617	0.729

**Table 7:** StructureNet average predictive performance on external validation datasets.

Model	MSE	MAE	R^2^	PCC	AUC
StructureNet_1_	3.040	1.326	0.216	0.441	0.678
StructureNet_2_	1.765	1.071	0.621	0.788	0.775
StructureNet_3_	0.823	0.610	0.525	0.724	0.828

**Table 8:** Comparison of existing structure-based graph neural network models for binding affinity prediction [22].

Machine Learning Model	Binding Affinity Dataset	MSE	PCC
StructureNet	PDBBind v2020 Refined Set (*n* = 5316)	1.991	0.682
MPNN (PL)	PDBBind v2019 Hold-out (*n* = 3386)	1.512	0.645

**Table 9:** Comparison of successful deep learning models for binding affinity prediction [5,31,32,33,34,35].

Name	Feature Type(s)	Dataset	MSE	MAE	PCC
StructureNet	Structure	PDBBind v2020 Refined Set	1.964	1.125	0.683
StructureNet	Structure	PDBBind v2016 Core Set	2.013	1.094	0.749
StructureNet	Structure	PDBBind v2013 Core Set	2.356	1.171	0.701
Pafnucy	Structure, Sequence	PDBBind v2013 Core Set	1.62	N/A	0.70
DeepBindRG	Structure, Interaction	PDBBind v2013 Core Set	3.302	1.483	0.639
LigityScore	Structure, Interaction	PDBBind v2013 Core Set	2.809	1.335	0.713
LigityScore	Structure, Interaction	PDBBind v2016 Core Set	2.277	1.224	0.725
SIGN	Structure, Interaction	PDBBind v2016 Core Set	1.836	1.027	0.797
GraphBAR	Structure, Interaction	PDBBind v2016 Refined Set	1.694	1.368	0.662
HNN-denovo	Structure, Sequence, Interaction	PDBBind v2019 Refined Set	0.922	N/A	0.840

**Table 10:** Predictive performance of hybridizations between StructureNet and sequence- and interaction-based models. Two unique model versions (V1 and V2) were developed for both combinations of structural data with sequence and interaction data.

Model	MSE	MAE	R^2^	PCC	AUC
Structural + Sequence − V1	1.701	1.025	0.530	0.743	0.761
Structural + Sequence − V2	1.662	1.010	0.564	0.759	0.769
Structural + Interaction − V1	1.680	1.018	0.561	0.751	0.767
Structural + Interaction − V2	1.658	1.002	0.582	0.772	0.774

**Table 11:** StructureNet predictions for an exemplary complex (PDB ID: 4g8n) and its ten conformers generated from MD simulations. The conformer with the most accurate prediction is bolded.

Time Interval (ps)	Predicted Binding Affinity	Experimental Binding Affinity
0	5.846	7.2
500	5.882	7.2
**1000**	**5.908**	**7.2**
1500	5.461	7.2
2000	5.461	7.2
2500	5.399	7.2
3000	5.305	7.2
3500	4.964	7.2
4000	5.482	7.2
4500	5.695	7.2
5000	5.130	7.2

**Table 12:** StructureNet predictions vs. BINANA interaction calculations for an exemplary protein–ligand complex (PDB ID 4cig).

Complex Conformation	Predicted Binding Affinity	Experimental Binding Affinity	Total Protein–Ligand Interactions
Reference structure	5.496	3.67	5
Selected conformer	4.929	3.67	1

## Data Availability

StructureNet was developed using Python and can be tested through this GitHub repository: “https://github.com/sivaGU/StructureNet (accessed on 27 January 2025). The files in this repository include the code for StructureNet’s deep learning model, as well as StructureNet’s demo webpage: “https://structurenet-9iajem6ixrcj22nft7msen.streamlit.app/ (accessed on 1 April 2025)”. The File S1.zip and File S2.zip are located in https://github.com/sivaGU/StructureNet (accessed on 27 January 2025).

## Supplementary Materials

The following supporting information can be downloaded at https://www.mdpi.com/article/10.3390/bioengineering12050505/s1. The Supporting Information is available free of charge. The PDF document in the Supporting Information folder contains the tables and figures listed below, as well as locations for the XLSX and ZIP files. Table S1: Binding affinity predictions for the general and refined sets (XLSX). Table S2: Binding affinity predictions for the DUDE-Z dataset (XLSX). Table S3: Binding affinity predictions from hybridized models on mebendazole, sunitinib, and sulindac (XLSX). Figure S1: All violin plots from the DUDE-Z dataset binding affinity predictions (ZIP). Figure S2: All receiver operating characteristic (ROC) curves from the DUDE-Z dataset binding affinity predictions (ZIP). Figure S3: All BINANA visualization calculations used to check intermolecular interaction trends (ZIP). Table S4: Binding affinity predictions (refined set) from ablation study (XLSX). Table S5: Protein–ligand interaction calculations from BINANA for use with conformer complexes (XLSX). Table S6: Binding affinity predictions and scatter plots for the ten protein–ligand complexes used in MD simulations and their conformers (XLSX). File S1.zip: PDB files for the PDBBind v2020 refined set (ZIP). File S2.zib: PDB files for the PDBBind v2020 general set (ZIP).
