# Supplementary material for: StructureNet: Physics-Informed Hybridized Deep Learning Framework for Protein–Ligand Binding Affinity Prediction
Source: Bioengineering (Basel). 2025 May 10;12(5):505. doi: 10.3390/bioengineering12050505 (PMC12109334; doi:10.3390/bioengineering12050505)
Supplement: Supplementary file 1 [file bioengineering-12-00505-s001.zip › Supporting Information.pdf]

Supporting Information for StructureNet: Structure-Based Protein-Ligand Binding Affinity Prediction with a Novel Hybridized Deep Learning Framework.

Arjun Kaneriya[1], Madhav V. Samudrala[2], Harrish Ganesh[3], James Moran[4], Somanath Dandibhotla[5], Sivanesan Dakshanamurthy[6,*]

1. *College of William and Mary, William and Mary, Williamsburg, VA 23185, USA*
2. *College of Arts and Sciences, The University of Virginia, Charlottesville, VA, 22903, USA*
3. *VCU Life Sciences, Virginia Commonwealth University, Richmond, VA 22043, USA*
4. *College of Arts and Sciences, Georgetown University, Washington, DC 20057, USA*
5. *College of Engineering and Computing, George Mason University, Fairfax, VA 22030, USA*
6. *Department of Oncology, Lombardi Comprehensive Cancer Center, Georgetown University Medical Center, Washington, DC 20007, USA*

*Email: sd233@georgetown.edu

Table of Contents

List of Tables

Table S1. Binding Affinity Predictions for the PDBBind v.2020 General and Refined Sets.

Table S2. DUDE-Z Dataset Binding Affinity Predictions.

Table S3. Binding Affinity Predictions of Drugs from Hybridized Models.

Table S4. Binding Affinity Predictions on PDBBind v.2020 Refined Set from Ablation Study.

Table S5. Protein-ligand interaction calculations from BINANA for use with conformer ensembles.

Table S6. Binding affinity predictions and scatter plots for the ten protein-ligand complexes used in MD simulations and their conformers.

List of Figures

Figure S1. Violin plots from the DUDE-Z dataset binding affinity predictions.

Figure S2. Receiver operating characteristic (ROC) curves from the DUDE-Z dataset binding affinity predictions.

Figure S3. All protein-ligand complex visualizations from the BINANA online software used in intermolecular interaction trend comparison.

List of Files

File S1. The hydrogenated PDBBind v2020 refined set of protein-ligand complexes used to develop and evaluate StructureNet.

File S2. The hydrogenated PDBBind v2020 general set of protein-ligand complexes used to develop and evaluate StructureNet.

Table S1. Binding Affinity Predictions for the PDBBind v.2020 General and Refined Sets.

This is provided as an Excel file in the Supporting Information folder, Table S1.xlsx

Table S2. DUDE-Z Dataset Binding Affinity Predictions

This is provided as an Excel file in the Supporting Information folder, Table S2.xlsx

Table S3. Binding Affinity Predictions of Drugs from Hybridized Models

PDB Code	Drug	StructureNet	STR+SEQ	STR+INT	Experimental
7odn	mebendazole	5.62	5.16	5.16	5.14
6nfz	sunitinib	8.07	7.62	7.65	7.8
3g0f	sunitinib	7.66	7.61	7.52	7.66
3rx3	sulindac	6.44	6.69	6.68	6.53
2kaw	sulindac	5.09	5.18	5.14	4.97
4ks8	sunitinib	5.92	5.83	5.82	5.62
4qmz	sunitinib	6.11	6.65	6.67	6.47
3u2c	sulindac	6.16	6.32	6.34	6.53
4agd	sunitinib	8.08	8.34	8.31	8.41
6jok	sunitinib	9.16	8.50	8.72	9.1
4wev	sulindac	5.37	5.71	5.68	5.57
6ng0	sunitinib	8.29	8.42	8.37	8.19

Table S4. Binding Affinity Predictions on PDBBind v.2020 Refined Set from Ablation Study.

Metric→ Feature Removed↓	PCC	MSE	MAE	R^2	AUC
**Baseline**	0.683	1.964	1.125	0.466	0.743
**Coordinates**	0.679	2.046	1.143	0.462	0.751

**Atomic #**	0.665	2.199	1.179	0.402	0.724
**Total Degree**	0.665	2.111	1.161	0.443	0.747
**Hydrophobicity**	0.669	2.093	1.196	0.448	0.749
**HBA**	0.666	2.103	1.161	0.445	0.749
**Residue Encoding**	0.663	2.121	1.164	0.440	0.751
**# Hydrogens**	0.665	2.108	1.153	0.443	0.746
**Mass**	0.664	2.116	1.159	0.442	0.744
**Voronoi**	0.576	2.531	1.279	0.333	0.717
**Element**	0.665	2.112	1.161	0.443	0.740
**HBD**	0.659	2.142	1.165	0.435	0.743
**Hybridization**	0.661	2.130	1.164	0.438	0.739
**Electronegativity**	0.660	2.139	1.166	0.436	0.750
**Spherical Harmonics**	0.662	2.177	1.167	0.443	0.748

Table S5. Protein-ligand interaction calculations from BINANA for use with conformer ensembles. This is provided as an Excel file in the Supporting Information folder, Table S5.xlsx

Table S6. Binding affinity predictions and scatter plots for the ten protein-ligand complexes used in MD simulations and their conformers. This is provided as an Excel file in the Supporting Information folder, Table S6.xlsx

Figure S1. All violin plots from the DUDE-Z dataset binding affinity predictions.

These are located in a zip file in the Supporting Information folder named Figure S1.zip containing multiple figures in PNG format.

Figure S2. All receiver operating characteristic (ROC) curves from the DUDE-Z dataset binding affinity predictions.

These are located in a zip file in the Supporting Information folder named Figure S2.zip containing multiple figures in PNG format.

Figure S3. All protein-ligand complex visualizations from the BINANA online software used in intermolecular interaction trend comparison. These are located in a zip file in the Supporting Information folder named Figure S3.zip containing multiple figures in PNG format.

File S1. The hydrogenated PDBBind v2020 refined set of protein-ligand complexes used to develop and evaluate StructureNet.
These are located in a zip file in the StructureNet GitHub repository, the link for which is provided in our manuscript and in our demo webpage.

File S2. The hydrogenated PDBBind v2020 general set of protein-ligand complexes used to develop and evaluate StructureNet.
These are located in a zip file in the StructureNet GitHub repository, the link for which is provided in our manuscript and in our demo webpage.
